# Calcium Metabolism, Immunity and Reproduction in Early Postpartum Dairy Cows

**DOI:** 10.3390/ani15142103

**Published:** 2025-07-16

**Authors:** Szilvia Kusza, Zoltán Bagi, Putri Kusuma Astuti, George Wanjala, Ottó Szenci, Árpád Csaba Bajcsy

**Affiliations:** 1 Centre for Agricultural Genomics and Biotechnology, University of Debrecen, 4032 Debrecen, Hungary; bagiz@agr.unideb.hu (Z.B.); kusumastuti@live.com (P.K.A.); 2 Doctoral School of Animal Science, University of Debrecen, 4032 Debrecen, Hungary; 3 Institute of Animal Sciences and Wildlife Management, University of Szeged, 6800 Hódmezővásárhely, Hungary; geog.wanjala@gmail.com; 4 Department of Obstetrics and Food Animal Medicine Clinic, University of Veterinary Medicine, 1078 Budapest, Hungary; szenci.otto@univet.hu; 5 Clinic for Cattle, University of Veterinary Medicine Hannover, Foundation, 30173 Hannover, Germany; csaba.bajcsy@tiho-hannover.de

**Keywords:** vitamin D_3_, gene expression, bovine placenta, immunity, calcium metabolism, feto-placental growth

## Abstract

Vitamin D is essential for calcium homeostasis, bone mineralization, immunity, and disease prevention. In a field study with Holstein-Friesian dairy cows, the impact of prepartum vitamin D_3_ treatment on early postpartum placental gene expression, focusing on calcium metabolism, feto-placental growth, and immune response, had been investigated. Eight multiparous cows were treated with 10 mL vitamin D_3_ (1 million IU cholecalciferol/mL) intramuscularly on day 273 of pregnancy, while eight others remained untreated and served as controls. Placental tissues were collected post-calving, and gene expression was analyzed using quantitative real-time PCR. Among 23 genes, 5 showed significant downregulation in the treated group: *CaBP-9k* (reduced by 88.1% from 32.80 ± 91.50 to 3.90 ± 8.54), *ESR1* (reduced by 95.7% from 7.89 ± 17.87 to 0.34 ± 0.34), *LHR* (reduced by 96.5% from 3.75 ± 5.45 to 0.13 ± 0.17), *NOD1* (reduced by 94.1% from 4.21 ± 7.00 to 0.25 ± 0.30), and *TLR1* (reduced by 99.7% from 24.80 ± 61.45 to 0.07 ± 0.08). These results suggest that vitamin D_3_ supplementation affects key pathways related to calcium transport, reproductive function, and immune response in the bovine placenta. These molecular changes may help to explain improved calcium homeostasis and reduced postpartum complications, offering insights into how targeted nutritional interventions can enhance reproductive efficiency in high-producing dairy cows.

## Simple Summary

The aim of this study was to investigate how prepartum vitamin D supplementation affects the function and gene expression of the placenta in dairy cows after calving. Vitamin D plays a key role in calcium metabolism and immune system function, which is particularly important for the health and productivity of cattle. Sixteen Holstein-Friesian cows were included in the study, with eight receiving a vitamin D injection, while the other eight did not. The results showed that vitamin D supplementation led to significant downregulation in the expression of several key genes involved in calcium transport, reproductive processes, and immune response. The study concluded that vitamin D supplementation can improve the reproductive health and overall well-being of dairy cows, contributing to enhanced milk production and more sustainable livestock farming practices.

## 1. Introduction

The dairy sector of the European Union produces the second largest output value in the agricultural industry. In 2016, European dairy farmers produced 168.3 million tons of milk, from which 97% originated from cattle [1]. The annual milk yield is projected to increase despite a declining dairy cattle population, indicating successful genetic selection for high milk yield. However, this intense genetic selection for production traits has created significant challenges for reproductive efficiency [2,3,4,5].

A substantial body of evidence indicates that high lactational productivity is negatively correlated with reproductive performance in dairy cattle [6,7,8,9]. This negative relationship has been extensively studied at both physiological and cellular levels, with numerous factors proposed as potential causes of decreased reproductive efficiency, including genetic, physiological, nutritional, and general management factors [8,10,11]. Understanding and addressing these complex fertility challenges requires multi-faceted approaches that consider the intricate interplay between various biological systems.

Nutritional factors appear to be particularly influential in reproductive performance [7], with vitamin D emerging as a critical component. Since the discovery of the role of vitamin D in preventing rickets in dairy calves [12], researchers have extensively explored the physiological roles and metabolic pathways of this fat-soluble vitamin. While initially associated with skeletal development, subsequent studies revealed the crucial role of vitamin D in regulating calcium (Ca) and phosphorus (P) homeostasis [13,14]. More recent investigations have uncovered its additional functions in cell differentiation and proliferation control, as well as in activation of the innate immune mechanisms [15,16].

The relevance of vitamin D for dairy cattle is particularly significant due to the metabolic demands of lactation. Lactating cows mobilize substantial amounts of Ca and P from circulation during milk synthesis, requiring approximately 2.8 g Ca and 1.7 g P for each kg of 4% FCM (Fat-Corrected Milk) produced. With a daily vitamin D requirement of 5000 to 10,000 IU, high-producing dairy cows demand more Ca and P for milk synthesis than their lower-producing counterparts [17,18]. When Ca and P intakes from feed absorption and bone resorption fail to meet output amounts, a negative homeostatic condition develops, leading to a mineral imbalance. This explains why high-producing dairy cows, especially multiparous animals, are more susceptible to subclinical hypocalcemia, which can progress to clinical parturient paresis (milk fever) in severe cases [19].

Recent genomic research has significantly advanced our understanding of disease mechanisms in dairy cattle. Kusza and Bagi [20] conducted a comprehensive genomic analysis of major bacterial pathogens causing bovine mastitis and lameness, highlighting the significant economic impact of these conditions on dairy production. They reported that mastitis affects up to 50% of dairy herds globally, leading to significant reductions in milk production and quality, with annual losses exceeding USD 2–3 billion through decreased milk yield, treatment costs, and premature culling.

Several preventive strategies are used to avoid milk fever. One of them involves the prepartum parenteral administration of vitamin D; however, its beneficial effects are not consistently clear [21]. Research has demonstrated that vitamin D supplementation improves reproductive health [22], reduces mastitis incidence [23], and decreases the risk of retained fetal membranes and metritis [24] in dairy cows. Additionally, vitamin D supplementation has been shown to increase pregnancy rate, resulting in a shorter calving interval in multiparous cows [24,25].

The molecular mechanisms by which vitamin D influences reproductive physiology involve numerous biological pathways and a complex network of genes directly or indirectly associated with vitamin D functions. For calcium signaling, Transient receptor potential channel type 6 (*TRPV6*) and Calbindin-D9k (*CaBP-9k*) play crucial roles in the active transport mechanisms of mammals, including the placenta and the uterus, suggesting their importance in establishing and maintaining pregnancy [10]. These genes support materno-fetal Ca^2+^ transport, which is critical for fetal Ca^2+^ homeostasis, bone growth, and development [26].

Regarding feto-placental growth, luteinizing hormone receptor (*LHR*) expression in granulosa cells of the dominant follicle is essential for luteal cell formation and function [27]. Simões et al. [28] found that the ovulatory capacity in cattle correlates with increased follicle diameter and the consequent upregulation of *LHR* isoform expression in granulosa cells. Estrogen signaling, mediated by intracellular receptors such as estrogen receptor 1 (*ESR1*) and estrogen receptor 2 (*ESR2*), is crucial for granulosa cell differentiation and *LHR* expression [29]. *ESR1* is considered the primary receptor in bovine follicular development [30] and is required for normal dominant follicle development in cattle [31].

The health and functionality of these reproductive systems are significantly influenced by metabolic hormones, particularly insulin-like growth factor 1 (*IGF1*), implicated in folliculogenesis, conception rates, and overall reproductive success [32]. *IGF1*, a significant component of the somatotrophic axis regulating metabolism and tissue growth, plays essential roles in cattle reproduction, including folliculogenesis [33,34], first-service conception rates [35], and early corpus luteum and embryonic development [33,36].

Immune function also plays a critical role in reproductive performance. Endometrial infections significantly contribute to poor reproductive outcomes in dairy cattle. Beyond serving as a mechanical barrier, endometrial cells produce various factors important in immune response. Toll-like receptors (*TLR*s) represent a well-recognized protein group that differentiates pathogens from commensals and mediates cytokine and chemokine production [37,38]. Research has identified numerous immunity-associated genes expressed in the endometrium of infertile cows, including *TLR4*, *NOS-2*, *IL1α*, *IL1β*, and *IL6* [39]. Cows with endometritis exhibit elevated expression of proinflammatory cytokines, such as interleukins *IL1β* and *IL6* [40].

Beyond these intrinsic factors, external environmental conditions significantly impact reproductive efficiency. Heat stress exacerbates reproductive inefficiencies by affecting feed intake and, consequently, milk yield and overall health [41]. Physiological responses to heat stress include elevated body temperature and altered hormone levels, resulting in a reduced conception rate and extended calving interval [42]. Understanding these stressors grows increasingly important as climate change threatens dairy productivity, necessitating adaptive management strategies to enhance resilience and reproductive performance [43].

In this complex interplay affecting reproductive performance, nutritional factors remain pivotal. The role of vitamin D extends beyond bone health. It is essential for metabolism modulation, immune system support, and overall reproductive health [44]. High-yielding dairy cows require substantial calcium and phosphorus to maintain metabolic balance during lactation, and deficiencies can lead to severe reproductive issues, including parturient paresis [45]. Administering vitamin D supplements has demonstrated enhanced fertility, reduced postpartum complication incidence, and improved overall reproductive metrics [46,47].

Despite extensive research on the importance of vitamin D in calcium metabolism and immune function [48], its specific effects on placental gene expression in dairy cattle remains incompletely understood. This knowledge gap presents an opportunity to better understand the molecular mechanisms, by which vitamin D supplementation might improve reproductive outcomes in high-producing dairy cows.

The goal of this study was to examine the expression profiles of 23 genes, grouped into three categories: calcium signaling, feto-placental growth and immune response. We hypothesized that a single intramuscular vitamin D_3_ treatment would differentially regulate these gene pathways in Holstein-Friesian dairy cows, thereby improving calcium homeostasis, reproductive function and immune responses during the periparturient period.

## 2. Materials and Methods

### 2.1. Sampling Details

Placental tissues from cotyledons of 16 randomly selected Holstein-Friesian cows of a large-scale Hungarian dairy cattle farm (latitude: 47.412961; longitude: 17.428376) were collected immediately after calving from well-developed caruncles to ensure standardization across all specimens and stored for this study. The cows were divided in two groups, treated and control. The treated group included multiparous (with at least two previous calvings) cows (*n =* 8), which received 10 mL of vitamin D_3_ in form of a single intramuscular treatment (vitamin D_3_ 1.000.000 I.E. ad us. vet. (1 million IU cholecalciferol/mL), CP-Pharma, Burgdorf, Germany) on day 273 of gestation, one week prior to expected calving date, as calculated from the farm average. The dosage was selected based on established veterinary protocols to achieve physiological levels without causing hypercalcemia. The control group featured *n* = 8, also mainly multiparous cows with at least two previous calvings, but two primiparous cows were also included. These animals had not received any vitamin D_3_ treatment before or during the experiment.

Selection and treatment of the animals in the study were performed during late summer and early autumn. Placental tissues were sampled within 12 h after calving. Immediately after sampling, tissue samples were placed in RNAlater (Ambion Inc., Austin, TX, USA) and stored at −20 °C until analysis.

### 2.2. RNA Extraction and qRT-PCR Analysis

Total RNA was extracted from placental tissue samples using a High Pure RNA Isolation Kit (Roche Diagnostics, Mannheim, Germany), and the concentrations were measured using a NanoDrop ND-1000 (Thermo Fisher Scientific, Waltham, MA, USA) instrument. Reverse transcription was performed with the use of the High Capacity cDNA Reverse Transcription Kit (Thermo Fisher Scientific, Waltham, MA, USA) as well as 200 ng of total RNA and storage at −20 °C, while a quantitative real-time polymerase chain reaction (qRT-PCR) was performed with an ABI 7300 Real-Time PCR System (Applied Biosystems Inc., Foster City, CA, USA) with SYBR Green Master Mix (Applied Biosystems, Foster City, CA, USA) with a 3 min denaturation, followed by 50 cycles of 95 °C for 15 s, 60 °C for 20 s and 72 °C for 15 s. High-resolution melting analysis was performed for each run. Primers were designed by the Primer Express v3.0.1. program for the genes of interest (GOI) and the housekeeping gene (HG) (Table 1). A primer concentration of 600 nM was used in the reactions.

### 2.3. Statistical Analysis

The Pfaffl method [49] was used to examine relative gene expression values after they had been adjusted to *GAPDH* as the housekeeping gene. With the aid of the LinReg PCR version 2017.0 software [50], primer efficiency was determined. Statistical Package for the Social Sciences (SPSS Version 26; IBM Corp., Armonk, NY, USA) was used to perform statistical analysis. The two groups were compared using an independent-sample *t*-test and Mann–Whitney U test, while the normality assumption was assessed using a Shapiro–Wilk test. The assessment of homoscedasticity was performed using Levene’s test. To compare differences between the two groups, the Mann–Whitney U test and independent-sample *t*-test were used. An independent-sample *t*-test was used when Levene’s test gave no significant differences (*p* > 0.05); otherwise, the non-parametric Welch *t*-test was applied. The level of significance was set to *p* < 0.05.

## 3. Results

**Figure 1:**
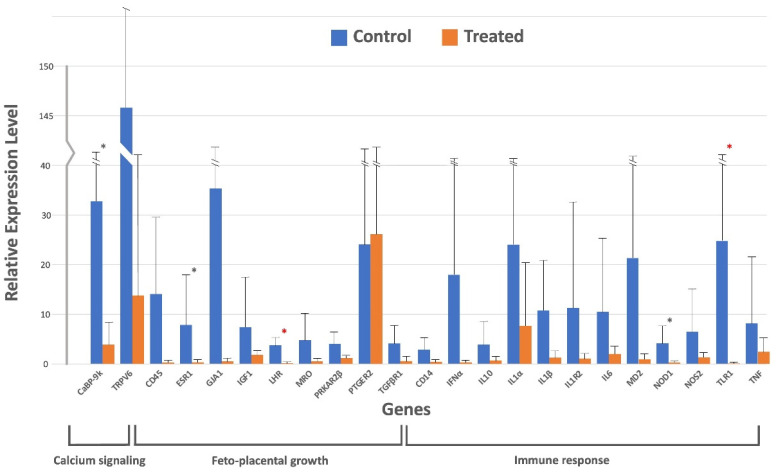
Real-time qPCR analysis for 23 genes related to calcium signaling, feto-placental growth, and immune response in the placenta of early postpartum Holstein-Friesian cows after a single prepartum intramuscular treatment with 10 million IU cholecalciferol. Fold change was calculated by the change in expression at a given time point relative to the untreated control and normalized by change in the *GAPDH* housekeeping gene, presented as mean ± SD for fold changes. ∗ indicates one-tailed significance level, and ∗ indicates two-tailed significance level. Means are presented by bars and SDs by upper closed lines.

The relative gene expression using the Pfaffl method [49] using *GAPDH* as the housekeeping gene is presented in Table 2 and visualized in Figure 1.

While most genes (18 genes: *TRPV6*, *CD45*, *GJA1*, *IGF1*, *MRO*, *PRKAR2β*, *PTGER2*, *TGFβR1*, *CD14*, *IFNα*, *IL10*, *IL1α*, *IL1β*, *IL1R2*, *IL6*, *MD2*, *NOS2*, and *TNF*) showed no significant variation in relative expression between the treated and control animals, significant (*p* < 0.05) downregulation of a few genes was observed in each category. For example, *CaBP-9k* was significantly downregulated (*p* < 0.05) under calcium signaling, whereas *ESR1* and *LHR* were significantly (*p* < 0.05) downregulated under feto-placental growth, and *NOD1* and *TLR1* were also significantly (*p* < 0.05) downregulated under immunological response.

For the calcium signaling gene, the relative gene expression of *CaBP-9k* was 32.80 ± 91.50 in the control group and downregulated to 3.90 ± 8.54 in the treated group. For the feto-placental growth, the expression of *ESR1* in the control group was 7.89 ± 17.87 and it was significantly downregulated in the treated group to the value of 0.34 ± 0.34, as was also the case for the *LHR* gene, with values of 3.75 ± 5.45 and 0.13 ± 0.17, for both the control and the treated group. Finally, for the immunological response gene group, both *NOD1* and *TLR1* genes were less expressed in the treated group, giving 0.25 ± 0.30 and 0.07 ± 0.08, respectively, compared to the control group, with values of 4.21 ± 7.00 and 24.80 ± 61.45, respectively.

## 4. Discussion

The possible effect of vitamin D supplementation on the expression of placental genes responsible for calcium signaling, feto-placental growth and immune response in heavily pregnant Holstein-Friesian cattle is presented. The influence of vitamin D on the health of both the dam and the calf pre- and postpartum cannot be overstated. Vitamin D is a pleiotropic secosteroid hormone that is important for health and disease prevention. The actions of vitamin D are mediated by the vitamin D receptor that binds the biologically active form of vitamin D [1,25-dihydroxycholecalciferol or 1,25(OH)_2_D] to induce both transcriptional and non-genomic responses. Vitamin D has a well-known classical function in calcium uptake and bone metabolism, but more recent works highlight the importance of the non-classical actions of vitamin D in a variety of cell types [24,51]. These actions include modulation of the innate and adaptive immune systems and regulation of cell proliferation.

Several research works have elucidated that the most critical period for dairy cows is the transition period, represented by a 3–4-week period prior to and after calving [51,52,53]. This interval is characterized by hampered immunity [53], inadequate energy intake [54], and an increased rate of fetal growth together with increased milk synthesis resulting in high demands for calcium [55]. Inadequate available calcium peripartum may lead to subclinical hypocalcemia, and in severe cases even its clinical form, milk fever, may develop. Practically, it is almost impossible to avoid subclinical hypocalcemia since extracellular calcium declines simultaneously with the intensification of milk synthesis that starts prior to calving in all pregnant dairy heifers and cows. In particular, high-producing, aged multiparous cows are susceptible to developing a significant decline in their blood calcium concentration, which occasionally results in critically low blood Ca^2+^ values that may lead to the appearance of clinical signs of parturient paresis. However, the severity of hypocalcemia can be reduced through several methods; one such option is preventive intramuscular treatment with vitamin D_3_.

These observations align with research by Gráff et al. [56], who examined the relationship between body condition score (BCS) and reproductive parameters in Holstein-Friesian cattle. They found that extreme body condition loss in early lactation can directly affect reproductive performance, including the number of days from calving to first service and conception. Metabolic stress experienced during this transition period has direct implications for vitamin D metabolism and calcium homeostasis, potentially explaining why strategic vitamin D supplementation during this critical window may improve reproductive outcomes. Their findings indicated that cows with BCS 3.5 or higher at calving and those maintaining BCS above 3.0 exhibited the most favorable reproductive performance, suggesting an optimal metabolic state for both calcium metabolism and reproductive function.

Vitamin D metabolism changes significantly during pregnancy in mammals. Maternal plasma levels of bioactive vitamin D (1α,25-(OH)_2_D) increase above those observed during the non-pregnant status but may exponentially increase in the late stages of gestation [57]. The concentration of placental vitamin D metabolites (1α,25-(OH)_2_D) determines the transcriptional changes/expression of placental genes within the placenta through vitamin D receptors and other relevant receptors. In humans, it is believed that placental vitamin D may regulate gene pathways vital for placental functions, fetal growth, and the postnatal health of the offspring. The effect of vitamin D on placental gene expression is unknown but it is thought to influence the epigenetic regulation of gene expression in humans [58], suggesting its importance in pregnancy health [59]. A study found that pregnant cattle given vitamin D in the form of calcidiol (25(OH)D) during the last three weeks of gestation had a higher pregnancy rate up to 55%, shorter median days open, and lower retained placenta rates [24]. While interpreting results in the present study, three assumptions are made: (a) the expression profile will be influenced by both the diet and the administered vitamin D preventive treatment, (b) any significant expression in treated cows is directly linked to the injected exogenous vitamin D, and (c) treatment with exogenous vitamin D raised the circulating vitamin D level in the animals to sufficiency (not a toxic level) and had a direct influence on targeted placental gene expression at the mRNA level.

The role of vitamin D in modulating immune response, particularly through the downregulation of proinflammatory genes like *NOD1* and *TLR1* observed in our study, may provide a mechanistic explanation for the reduced incidence of mastitis and improved reproductive health following vitamin D supplementation reported in previous studies [53]. The presence of *CABP-9k* and *TRPV6* genes in bovine fetal membranes suggests placental calcium metabolism, which is essential for reproductive health [59]. We observed a significant (*p* < 0.05) downregulation of *CABP-9k* in the treated animals compared to the controls, suggesting that a higher concentration of vitamin D downregulates *CABP-9k*. However, the expression levels of *TRPV6* genes between the two groups were not significantly different. Many studies suggest that both *CABP-9k* and *TRPV6* genes are actively involved in the transportation of Ca^2+^ from the dam’s organismus to the fetus, although the study in [26] showed that their gene expression was not correlated but rather activated at different and non-overlapping times [60]. Some studies have proposed that the *CABP-9k* gene is induced by vitamin D [61,62]. Although the mechanisms for vitamin D transportation to the placenta and placental vitamin D metabolism are unknown, particularly in dairy cows, we hypothesize that intramuscular treatment with a vitamin D_3_ preparation may have resulted in increased concentrations of vitamin D in the placenta, which in turn may have increased rates of calcium and phosphorus metabolism to sufficiency, limiting mobilization of these minerals from the bones and resulting in the downregulation of the *CABP-9k* gene [26,57,63]. Schulz [64] observed that maternal vitamin D sufficiency significantly reduced the expression of angiogenic genes at the mRNA level in humans.

Furthermore, the expression of some feto-placental growth genes (*CD45*, *GJA1*, *IGF1*, *MRO*, *PRKAR2β*, *PTGER2* and *TGFβR1*) in the treated animals did not significantly differ from the controls. However, the relative expression of *ESR1* and *LHR* in the controls was significantly (*p* < 0.05) higher than in the treated cows. Genome-wide studies in cattle have significantly associated most of the above-mentioned genes including *IGF1*, *CD45*, and *TGFβR1* with pregnancy maintenance and fetal growth [10], whereas a study in which *PRKAR2β* was knocked out in mice resulted in failure of fetal maturation [65], suggesting the role of this gene in fetal maturation. Other studies have also indicated that the knockout of the *ESR1* gene is associated with infertility in females; in addition, the gene is also vital in the regulation of growth and expansion of the placental vascular network [66]. Based on our results, we interpret that all the animals were vitamin D-sufficient at the terminal phase of gestation and an additional vitamin D treatment induced the expression of *ESR1* and *LHR,* both of which play a vital role in the fertility of an individual, and supplementation of vitamin D can modulate the hormone receptor signaling pathway. The role of vitamin D in cell proliferation is not disputable. Three to four weeks prior to the expected calving date, the fetal growth rate is at its peak; hence, adequate vitamin D in the dam’s plasma and placenta would ensure the fetus attains the genetic birth weight. Several studies using model animals like mice suggest that the risk of stillbirth is higher when the mother is vitamin D-deficient [67]. Postpartum complications were also reported in mice [68], and this can be extrapolated to dairy cattle as well. Fetal mortality is a major cause of economic losses in dairy cattle considering the increased calving interval as estimated by Diskin and Morris [69]. Gene expression of feto-placental growth in dairy cattle following a treatment with cholecalciferol is not known, but the expression levels in the present study indicated that supplementation with an additional recommended dosage can shift placental gene expression. In studies conducted by Vestergaard [70] and Magiełda-Stola [71] on humans, authors reported that vitamin D deficiency was significantly associated with fetal growth restriction and preeclampsia, and a similar situation can be extrapolated to dairy cattle. In our study, higher levels of significant relative expression levels of *ESR1* and *LHR* genes could be an indication of the beneficial functions of vitamin D in the reproductive efficiency of the animal.

Similar to the preceding group of genes, the expression levels of immune response genes also link vitamin D to immunity response. In the present study, the relative expression of all immune response genes in treated animals was not significantly different from that of the untreated ones, except the *NOD1* and *TLR1* genes, which were significantly downregulated. Accumulating evidence suggests that vitamin D beneficially reduced *TLR1* and *NOD1* in Crohn’s disease [72]. Maternal vitamin D signaling is believed to be vital in boosting the innate immunity indices as well as suppressing the proinflammatory adaptive immunity in cattle [14,16,51], whereas placental vitamin D is thought to be vital in the regulation of placental inflammation [73]. The experiment conducted by Liu et al. [73] reported that immune response genes were significantly upregulated in mice challenged with lipopolysaccharide (LPS), especially in wild-type placentas, whereas anti-inflammatory regulators such as IL-10 were downregulated in placentas lacking either trophoblastic Cyp27b1 or VDR, indicating a dysregulated immune response. Ex vivo treatment of mice placentas with the substrate *Cyp27b1* showed that 25-hydroxyvitamin D_3_ significantly suppressed the expression of most of the immune response genes. Therefore, the indifferent expression of most of the immune response genes in the present study could have been due to the suppression of proinflammatory adaptive immunity induced by diet composition. The mechanisms by which vitamin D around calving enhances immunity are widely studied and reviewed [16,52,74,75].

The influence of vitamin D on calcium metabolism, fetal growth, pregnancy maintenance, immunity response and other functions, whose discussion is beyond the scope of the present study, is undebatable. The level of milk yield and calving interval exert a critical economic impact on a dairy farm. The optimal calving interval proposed by many researchers and practitioners working with dairy cattle is 380 days [76], which seems to be way below the current status in the Hungarian dairy sector, which exceeds 423 days [77]. Efforts to reduce the calving interval in Hungary below 400 days are ongoing, the success of which would lead to a significant reduction in general production costs. One major factor in optimizing the calving interval is to improve the nutritional and health status of dairy cows during the transition period. In this respect, an important component is the reduced or complete loss of muscle activity in the early postpartum uterus. Several factors can be found to be responsible for this, such as hypocalcemia, possible overstretching of a certain amount of uterine muscle fibers due to twin pregnancy, energy deficiency, subclinical acidosis and many other metabolic, reproductive or related disorders. If postpartum uterine activity could be adequately enhanced using proper uterotonic treatment protocols, the processes and, thereby, the speed of involution may improve. Tissue responses include effects on hormone secretion, modulation of immune responses, and control of cellular proliferation and differentiation [74].

## 5. Conclusions

This study demonstrates that prepartum intramuscular vitamin D_3_ supplementation significantly alters the placental expression of key genes involved in calcium metabolism (*CaBP-9k*), reproductive signaling (*ESR1*, *LHR*), and immune response (*NOD1*, *TLR1*) in multiparous Holstein-Friesian dairy cows. These molecular changes may contribute to improved calcium homeostasis, enhanced reproductive function, and modulated inflammatory responses during the periparturient period. Our findings provide insights into how targeted vitamin D supplementation could support transition health and reproductive efficiency in high-producing dairy cattle, highlighting its potential as a practical nutritional intervention in herd management.

## Figures and Tables

**Table 1:** The sequences of the primers of the genes of interest (GOI) and the housekeeping gene (HG) as designed for the study.

	Main Role	Gene	Forward (5′-3′)	Reverse (5′-3′)
HG		*GAPDH*	CCACGAGAAGTATAACAACACC	GTCATAAGTCCCTCCACGAT
GOI	Calcium signaling	*CaBP-9k*	TCTCCAGAAGAACTGAAGGGC	CCAACACCTGGAATTCTTCG
		*TRPV6*	CAAGGAGCCCATGACATCTGA	CAGGGCTTTCACGAGGTTCA
	Feto-placental growth	*CD45*	CTCGATGTTAAGCGAGAGGAAT	TCTTCATCTTCCACGCAGTCTA
		*ESR1*	CCAACCAGTGCACGATTGAT	TTCCGTATTCCGCCTTTCAT
		*GJA1*	GTCTTCGAGGTGGCCTTCTTG	AGTCCACCTGATGTGGGCAG
		*IGF1*	AGCAGTCTTCCAACCCAA	AGATGCGAGGAGGATGTG
		*LHR*	AAACTTGCCAACAAACGA	ATAGCAAGTCTTGTCCAGGA
		*MRO*	CCCACTTACAGGACAGGAATCC	TGGAAGCTGTAGTCCTTGCTTTG
		*PRKAR2β*	GGGCATTCAACGCTCCAGTA	CTGGATTCAGCATCATCTTCTTCTT
		*PTGER2*	GTTCCACGTGTTGGTGACAG	ACTCGGCGCTGGTAGAAGTA
		*TGFβR1*	CAGGTTTACCATTGCTTGTTCA	TGCCATTGTCTTTATTGTCTGC
	Immune response	*CD14*	GGGTACTCTCTGCTCAAGGAAC	CTTGGGCAATGTTCAGCAC
		*IFNα*	AGAGCCTCCTGGACAAGCTAC	CATGACTTCTGCTCTGACAACC
		*IL10*	TACTCTGTTGCCTGGTCTTCCT	AGTAAGCTGTGCAGTTGGTCCT
		*IL1α*	AGAGGATTCTCAGCTTCCTGTG	ATTTTTCTTGCTTTGTGGCAAT
		*IL1β*	GAGGAGCATCCTTTCATTCATC	TTCCTCTCCTTGTACGAAGCTC
		*IL1R2*	ATCCCATGTAAGGTGTTTCTGG	TGACAGGATCAAAAATCAGTGG
		*IL6*	ATGACTTCTGCTTTCCCTACCC	GCTGCTTTCACACTCATCATTC
		*MD2*	GGGAAGCCGTGGAATACTCTAT	CCCCTGAAGGAGAATTGTATTG
		*NOD1*	GTCACTCACATCCGAAACACTC	CCTGAGATCCACATAAGCGTCT
		*NOS2*	GGACAGTAAAGACGTCTCCAGA	TATGGTCAAACTTTTGGGGTTC
		*TLR1*	CCCACAGGAAAGAAATTCCA	GGAGGATCGTGATGAAGGAA
		*TNF*	ACTCAGGTCCTCTTCTCAAGCC	ATGATCCCAAAGTAGACCTGCC

**Table 2:** Relative gene expression of 23 genes related to calcium homeostasis, feto-placental development, and immune response in bovine placenta within 12 h postpartum due to a single intramuscular prepartum treatment with 10 million IU cholecalciferol on day 273 of pregnancy.

Gene	Control Group (Mean ± SD)	Treated Group (Mean ± SD)
*CaBP-9k*	32.80 ± 91.50	3.90 ± 8.54
*CD14*	2.95 ± 5.48	0.41 ± 0.45
*CD45*	14.08 ± 29.19	0.28 ± 0.38
*ESR1*	7.89 ± 17.87	0.34 ± 0.34
*GJA1*	35.37 ± 101.87	0.49 ± 0.76
*IFNα*	17.96 ± 51.38	0.33 ± 0.32
*IGF1*	7.40 ± 17.22	1.81 ± 1.85
*IL10*	3.94 ± 8.49	0.67 ± 0.68
*IL1α*	24.06 ± 51.76	7.70 ± 20.01
*IL1β*	10.84 ± 21.92	1.26 ± 2.02
*IL1R2*	11.34 ± 31.86	1.08 ± 1.32
*IL6*	10.49 ± 24.86	2.03 ± 2.49
*LHR*	3.75 ± 5.45	0.13 ± 0.17
*MD2*	21.34 ± 60.14	0.92 ± 0.97
*MRO*	4.78 ± 10.24	0.49 ± 0.61
*NOD1*	4.21 ± 7.00	0.25 ± 0.30
*NOS2*	6.47 ± 15.92	1.32 ± 1.90
*PRKAR2β*	4.09 ± 6.66	1.18 ± 1.40
*PTGER2*	24.11 ± 68.56	26.36 ± 75.45
*TGFβR1*	4.12 ± 7.21	0.53 ± 0.71
*TLR1*	24.80 ± 61.45	0.07 ± 0.08
*TNF*	8.19 ± 22.27	2.43 ± 5.19
*TRPV6*	145.64 ± 430.47	13.89 ± 42.23

## Data Availability

The data presented in this study are available on request from the corresponding author.
